# *Withania coagulans* Fruit Extract Reduces Oxidative Stress and Inflammation in Kidneys of Streptozotocin-Induced Diabetic Rats

**DOI:** 10.1155/2014/201436

**Published:** 2014-09-14

**Authors:** Shreesh Ojha, Juma Alkaabi, Naheed Amir, Azimullah Sheikh, Ahmad Agil, Mohamed Abdelmonem Fahim, Abdu Adem

**Affiliations:** ^1^Department of Pharmacology and Therapeutics, College of Medicine and Health Sciences, United Arab Emirates University, P.O. Box 17666, Al Ain, UAE; ^2^Department of Internal Medicine, College of Medicine and Health Sciences, United Arab Emirates University, P.O. Box 17666, Al Ain, UAE; ^3^Department of Pharmacology and Neurosciences Institute, School of Medicine, University of Granada, 18012 Granada, Spain; ^4^Department of Physiology, College of Medicine and Health Sciences, United Arab Emirates University, P.O. Box 17666, Al Ain, UAE

## Abstract

The present study was carried out to investigate the changes in oxidative and inflammatory status in streptozotocin-induced diabetic rat's kidneys and serum following treatment with *Withania coagulans*, a popular herb of ethnomedicinal significance. The key markers of oxidative stress and inflammation such as inflammatory cytokines (IL-1*β*, IL-6, and TNF-*α*) and immunoregulatory cytokines (IL-4 and IFN-*γ*) were increased in kidneys along with significant hyperglycemia. However, treatment of four-month diabetic rats with *Withania coagulans* (10 mg/kg) for 3 weeks significantly attenuated hyperglycemia and reduced the levels of proinflammatory cytokines in kidneys. In addition, *Withania coagulans* treatment restored the glutathione levels and inhibited lipid peroxidation along with marked reduction in kidney hypertrophy. The present study demonstrates that *Withania coagulans* corrects hyperglycemia and maintained antioxidant status and reduced the proinflammatory markers in kidneys, which may subsequently reduce the development and progression of renal injury in diabetes. The results of the present study are encouraging for its potential use to delay the onset and progression of diabetic renal complications. However, the translation of therapeutic efficacy in humans requires further studies.

## 1. Introduction

Diabetes, a rising epidemic throughout the world, has no signs of abatement and remains one of the most challenging health problems. People with diabetes suffer from the detrimental vascular which accounts for high morbidity and mortality [1]. Among several vascular complications, chronic renal failure and end stage renal diseases appear first and often associated with metabolic and hemodynamic alternations. The development and progression of diabetes and associated vascular complications are largely precipitated by chronic hyperglycemia-induced oxidative stress [2]. In addition to oxidative stress, immune-mediated low grade chronic inflammatory mechanisms have been demonstrated to play a significant role in pathogenesis of renal injury in long term diabetes [3]. Convincing number of studies demonstrates that oxidative stress and immune inflammatory processes intimately linked together causing renal damage through multiple mechanisms [3–6].

The management of diabetic renal complications is based on the approaches to delay the development and progression by keeping strict control of blood pressure or plasma glucose [7]. However, controlling the blood pressure and plasma glucose levels to prevent the renal complications is far from satisfactory [7]. This imperfection points to the need for newer therapeutic agents that have potential to target these intimately linked cascade; oxidative stress-inflammatory cytokine signaling and delay the progression and development of renal complications in diabetes [7]. Therefore, in search of newer therapeutic agents, medicinal plants considered as a major source of drug discovery from natural origin have been extensively explored [1].

Subsequently, many plant-derived natural products have the potential to be effective in diabetic renal complications by attenuating oxidative stress and proinflammatory and immunoregulatory cytokines [8–10]. The challenge is to identify the most promising compounds and evaluate their protective mechanism. The fruits of* Withania coagulans *belonging tofamily Solanaceae have received considerable attention for their benefits in chronic degenerative diseases including diabetes. The plant,* Withania coagulans*, commonly known as Indian Rennet, vegetable rennet (English), Panir dodi (Hindi), and Ning gu shui qie (Chinese), has been reported to possess a variety of ethnomedicinal uses [11]. The extract has shown potential activities, namely, anticancer [12], wound healing [13], immunomodulating [14], antihyperglycemic [15], and hypolipidemic [16] activities.

Despite several reports of its benefits in diabetes [11, 15, 17–19] and considering its potential to target the complex interplay of oxidative stress and inflammatory and immunoregulatory cytokines in diabetic renal complication it is worthwhile to study the effect of* Withania coagulans* in kidneys of streptozotocin- (STZ-) induced diabetes. In order to understand the mechanism, the present study examined the effect of* Withania coagulans *on antioxidant defense, lipid peroxidation, and immunoregulatory and proinflammatory cytokines.

## 2. Material and Methods

### 2.1. Chemicals

STZ, sodium citrate, citric acid, bovine serum albumin, 5-sulfosalicylic acid (SSA), naphthalene diamine dihydrochloride, sulphanilamide, phosphoric acid, HEPES ((4-(2-hydroxyethyl)-1-piperazineethanesulfonic acid), sucrose, 1,4-dithiothreitol (DTT), CHAPS 3-[(3-Cholamidopropyl)dimethylammonio]-1-propanesulfonate, sodium chloride, protease inhibitors, phenylmethylsulfonyl fluoride (PMSF), tween 20, sodium nitrate, 3,3,5,5′-Tetramethylbenzidine (TMB), glutathione (GSH) assay kit, and all other required chemicals, if not specified, were purchased from Sigma-Aldrich (Sigma Chemical Co., St. Louis, MO, USA). All chemicals used in the present study were of analytical grade. Malondialdehyde (MDA) assay kit was purchased from Northwest Life Science Specialties (WA, USA). Cytokines duo set ELISA kits were purchased from R&D Systems (Minneapolis, MN, USA). EnzChek myeloperoxidase (MPO) activity assay kit was purchased from Life Technologies (NY, USA).

### 2.2. Animals and Diet

Male Wistar rats (230 to 250 g) bred in the animal research facility of College of Medicine and Health Sciences, United Arab Emirates University, Al Ain, UAE, were used. The animals were housed under standard laboratory conditions (22 ± 2°C and 65 ± 5% humidity) and maintained on a 12-hour light/dark cycle. The animals had free access to food and water and were fed commercially available standard rat diet. A maximum of four rats were housed per cage and acclimatized to the laboratory conditions prior to the commencement of the experiment. The experimental protocols were approved by the Institutional Animal Ethics Committee of College of Medicine and Health Sciences (IAEC CMHS), United Arab Emirates University, Al Ain, UAE, and conducted according to the criteria outlined in the guide for the care and use of laboratory animals by the National Academy of Sciences.

### 2.3. Preparation of the* Withania coagulans *Aqueous Fruit Extract

A standard protocol was followed for the extraction of* Withania coagulans*. The fruits of* Withania coagulans *(0.28 g/100 mL) were soaked in distilled water overnight followed by a mechanical dispersion using a sterile cotton wood (Hardwood Products Company, Guilford, CT, USA) and filtration through cheese cloth. The dose of 10 mg/kg was selected based on a dose response pilot study in our laboratory. A total of five doses (0, 10, 125, 625, and 1250 mg/kg) were screened to find out the optimal dose following a dose response curve in a postprandial glucose test based dose response study. Five groups of six STZ diabetic rats each were fasted overnight and used in the experiment. Group I served as diabetic control and received vehicle (distilled water only). Rats of groups II, III, IV, and V received doses of 10, 125, 625, and 1250 mg/kg, respectively, of aqueous fruit extract suspended in distilled water. The level of baseline blood glucose was measured at 0 hr, followed by an oral administration of either distilled water (diabetic control group) or* Withania coagulans* extract. The rats were allowed to have free access to food and water. The blood samples were collected from tail vein at 1, 2, 3, and 4 hrs after giving the extract using an ACCU-CHEK performa glucometer. Among the doses studied, the dose of 10 mg/kg was found most potent in exhibiting the antihyperglycemic activity (results not shown). For further experiments, the dose of 10 mg/kg was chosen and a detailed study was performed.

### 2.4. Induction of Experimental Diabetes in Rats

A single dose of 60 mg/kg STZ was dissolved in freshly prepared citrate buffer (pH 4.5; 0.1 M) and injected intraperitoneally to induce diabetes. The age matched control rats received an equal amount of citrate buffer and were used along with the diabetes control group. Diabetes was confirmed by using Accucheck performa glucometer (Roche Diagnostics, NSW, Australia), after 48 hours of STZ injection. The rats having plasma glucose levels of >350 mg/dL were considered as diabetics and were used in the present study. The rats injected with STZ provide a relatively inexpensive and easily accessible rodent model that is not extremely obese and simulates the natural history and metabolic characteristics of patients with diabetes mellitus [20].

### 2.5. Experimental Design

**Figure 1:**
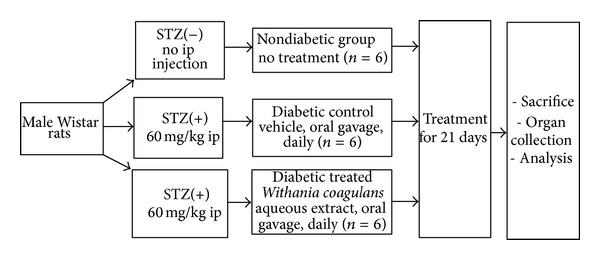
Schematic diagram of the experimental groups and treatment protocol.

The rats were divided into three experimental groups, each consisting of six rats. Group 1 served as nondiabetic controls group. The group 2 and 3 rats were four-month diabetic at the start of the experiment. Group 2 served as STZ-induced diabetic group; group 3 served as diabetic group treated orally with* Withania coagulans *(10 mg/kg/day b.w. for 3 weeks). The schematic representation of the experimental groups and treatment procedure are presented in Figure 1. During the experimental period, the body weight and blood glucose were determined at regular intervals. The blood glucose level was measured before treatment and after the 3-week treatment over a period of 4 h. At the end of the experimental period, rats were euthanized and the kidneys were removed and processed for the estimation of reduced glutathione (GSH), malondialdehyde (MDA), nitric oxide (NO), and cytokines (IL-1*β*, IL-4, IL-6, TNF-*α*, and IFN-*γ*) using the specific kits.

### 2.6. Preparation of Kidney Tissue Homogenate

The kidneys were removed, weighed, washed in ice-cold PBS, and minced into 2–5 mm fragments followed by homogenization using a polytron homogenizer (IKA Laboratory, Germany), with 5 volumes of ice-cold buffer containing 100 mM HEPES, pH 7.5, 10% sucrose, 10 mM DTT, 0.1% CHAPS, 150 mM NaCl, protease inhibitors tablet, and 1 mM PMSF. The samples were centrifuged at 10000 ×g for 10 min and the obtained supernatant was removed and stored at −80°C until the assessment of MPO activity and cytokines using ELISA kits.

### 2.7. Determination of Oxidative Stress Markers

The levels of GSH and MDA were determined using commercially available kits in serum and kidney. The level of NO was measured only in kidney tissues.

### 2.8. Estimation of Reduced Glutathione (GSH)

The GSH content in serum and kidney homogenate was estimated following manufacturer protocol of the assay kit. Briefly, the measurement of GSH uses a kinetic assay in which catalytic amounts (nmoles) of GSH cause a continuous reduction of 5,5-dithiobis (2-nitrobenzoic acid) to nitrobenzoic acid (TNB), and the glutathione disulfide (GSSG) formed was recycled by glutathione reductase and NADPH. The yellow color product, 5-thio-2-TNB, was measured spectrophotometrically at 412 within 5 min of 5,5-dithio-bis(2-nitrobenzoic acid) addition, against a blank with no homogenate. GSH concentration was expressed as *μ*M of GSH per milligram of tissue or per 0.01 mL of serum.

### 2.9. Estimation of Malondialdehyde (MDA)

The lipid peroxidation product, MDA, in the kidney homogenate from each group was measured using the MDA assay kit. Briefly, the assay is based on the reaction of MDA with thiobarbituric acid (TBA) to form a MDA-TBA adduct that absorbs strongly at 532 nm. Briefly, the deproteinated tissue sample was added to 1 M phosphoric acid and butylated hydroxytoluene in ethanol and then the mixture was heated at 60°C for 60 min. The suspension was cooled to room temperature and centrifuged at 10000 ×g for 2-3 min and the pink colored supernatant was taken for spectroscopic measurements at 532 nm for the assay of MDA. The concentration of MDA was expressed as *μ*M per 10 milligram of tissue or per 0.1 mL serum.

### 2.10. Assay of Myeloperoxidase (MPO) Activity

The chlorination assay for MPO activity in serum and kidney homogenate (ng/mg tissue wet weight) was performed in a microtiter plate using the EnzChek MPO activity assay kit. Briefly, 50 *μ*L of 2 × 3′-(*p*-aminophenyl) fluorescein working solution was added to 50 *μ*L of sample. The reaction mixture was then incubated in the dark at 37°C for 20 min. The fluorescence intensity of each sample was recorded at 485 nm excitation and 530 nm emission on a Perkin Elmer luminescence spectrofluorometer.

### 2.11. Estimation of Nitric Oxide (NO)

Accumulation of nitric oxide was used to determine the production of NO according to the Griess reagent (0.2% naphthylene diamine dihydrochloride and 2% sulphanilamide in 5% phosphoric acid) method. Briefly, 100 *μ*L of sample was mixed with an equal volume of Griess reagent and incubated at room temperature for 10–15 min. The absorbance at 492 nm was measured in an automated microplate reader (Tecan Group Limited, Männedorf, Switzerland). The nitrite concentration was quantitated using NaNO_2_ as standard and was expressed as micromolar concentrations of NO per mg tissue.

### 2.12. Determination of Proinflammatory Cytokines in Kidney

Enzyme immunoassay of IL-1*β*, IL-4, IL-6, TNF-*α*, and IFN-*γ* in kidney homogenate was performed by using commercial sandwich R&D duoset ELISA kit (Minneapolis, USA). Briefly, the wells of a 96-well microtiter plate were coated with respective primary antibody in phosphate buffer saline (PBS), (100 *μ*L/well), overnight at room temperature, washed with phosphate-buffered saline containing 0.05% Tween-20 (PBST), and then blocked with 1% bovine serum albumin in PBS for one hour. After washing, plates were incubated with serum, kidney homogenates, and respective standards for 2 hours. After washing with PBST, a detection antibody was added for 2 hours and 100 *μ*L of HRP was added for half an hour, after the washing. The TMB-ELISA substrate was added and the color intensity read at 450 nm with a microplate reader (Tecan Group Ltd., Männedorf, Switzerland). Cytokines levels were expressed as pg per milligram of tissue wet weight and per mL of serum.

### 2.13. Statistical Analysis

Data was analyzed statistically using SPSS 19.0 software. The means of the data are presented with the standard error mean (SEM). The results were analyzed using one-way ANOVA to determine the significance of the mean between the groups. Values of *P* < 0.05 were considered significant.

## 3. Results

### 3.1. Effect of* Withania coagulans* on Body Weight and Kidney to Body Weight Ratio

Table 1 shows the changes in body weight and the ratio of kidney/body weight in different experimental groups. There was a significant (*P* < 0.001) decrease in the body weight of rats administered STZ in comparison with rats of nondiabetic control group. Diabetic rats treated with* Withania coagulans *show a significant (*P* < 0.05) improvement in body weight when compared to diabetic control rats. Ratio of kidney/body weight is an index of renal hypertrophy and a significant (*P* < 0.001) increase in kidney/body weight indicates renal injury in STZ administered rats. However, treatment with* Withania coagulans *to the diabetic rats has significantly (*P* < 0.05) reduced renal hypertrophy as evidenced by reduction of kidney/body weight when compared to the diabetic control.

### 3.2. Effect of* Withania coagulans* on Blood Glucose, BUN, and Creatinine

**Figure 2:**
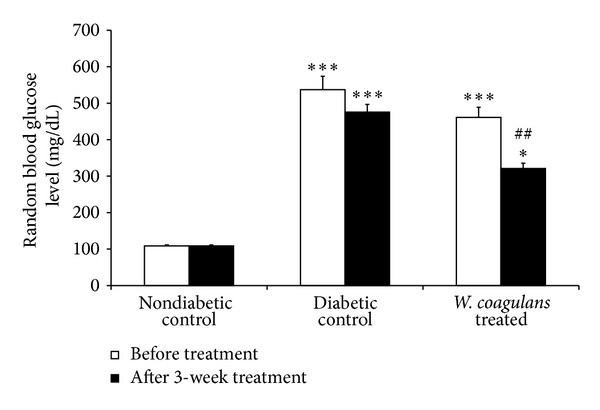
Effect of* Withania coagulans *on blood glucose level. The diabetic treated rats showed significant decrease in blood glucose levels compared to diabetic controls. Results are means ± SEM; *n* = 6 rats; *P < 0.05, **P < 0.01, ***P < 0.001 from nondiabetic controls; ^##^ *P* < 0.01, from diabetic controls.

The changes in the level of blood glucose and serum insulin in the rats of different experimental groups are represented in Figure 2. A significant (*P* < 0.001) and persistent rise in plasma glucose level was observed in STZ administered rats as compared with nondiabetic control group. However, a significant (*P* < 0.001) reduction was observed in the plasma glucose level of diabetic rats treated with* Withania coagulans *when compared to diabetic controls The BUN and creatinine levels were not different between the different groups (results not shown).

### 3.3. Effect of* Withania coagulans* on Glutathione

**Figure 3:**
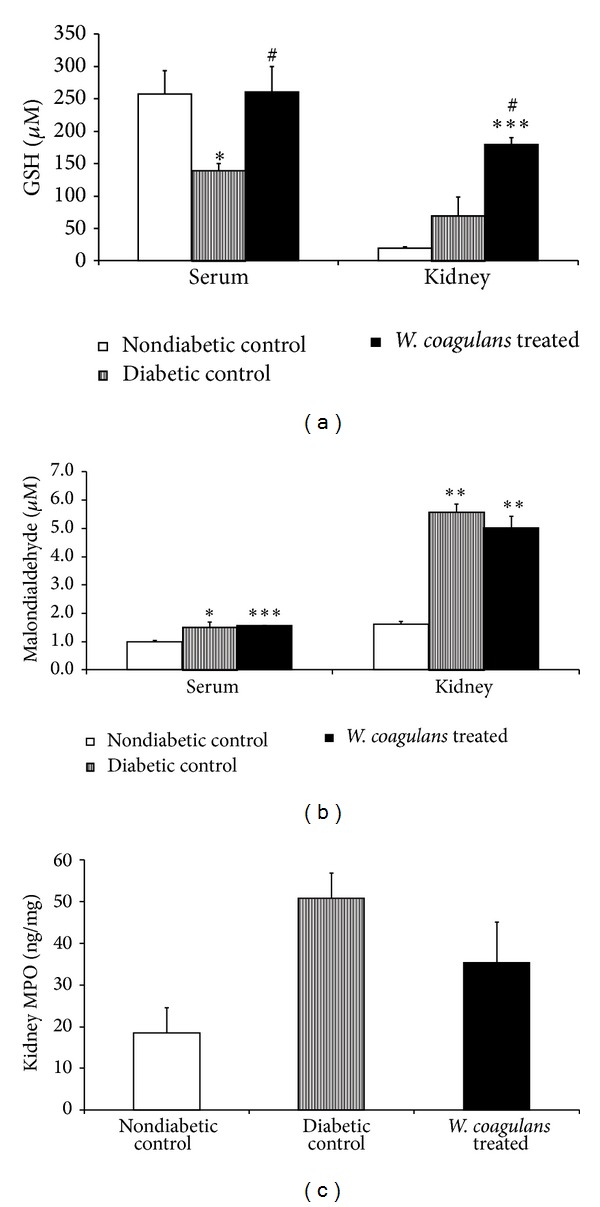
Effect of* Withania coagulans *on serum and kidney levels of (a) GSH, (b) MDA, and (c) MPO. Results are means ± SEM; *n* = 6 rats; *P < 0.05, **P < 0.01, ***P < 0.001 from nondiabetic controls; ^#^ *P*  0.05 from diabetic controls.

Animals administered STZ showed a significant (*P* < 0.05) decrease in the serum GSH level when compared to the nondiabetic control group (Figure 3(a)). However, no significant change in kidney GSH level was observed in diabetic rats when compared to the nondiabetic control group. Treatment with* Withania coagulans *extract significantly (*P* < 0.05) induced the level of GSH, both in serum and in kidney of diabetic rats when compared to diabetic control group (Figure 3(a)).

### 3.4. Effect of* Withania coagulans* Lipid Peroxidation

The rats administered STZ showed a significant increase in the MDA levels of serum (*P* < 0.05) and kidney (*P* < 0.001) as compared to the nondiabetic control group (Figure 3(b)). However, treatment with* Withania coagulans *has not reduced the level of MDA in serum and showed a slight nonsignificant decrease in the kidney compared to diabetic control group (Figure 3(b)).

### 3.5. Effect of* Withania coagulans* on MPO Activity

A modest but insignificant increase in MPO levels in kidney of the diabetic control group was observed when compared to non-diabetic control group (Figure 3(c)). However, treatment with* Withania coagulans *was found todecrease MPO levels in kidney as compared to the diabetic control group (Figure 3(c)). The decrease in MPO levels was not significant in any group.

### 3.6. Effect of* Withania coagulans* on Nitric Oxide

**Figure 4:**
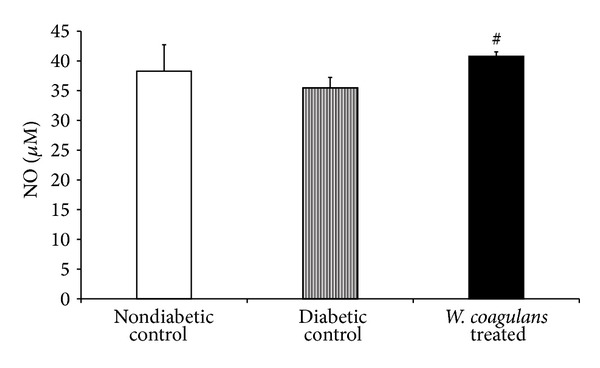
Effect of* Withania coagulans *on levels of NO in kidney. Results are means ± SEM; *n* = 6 rats; ^#^ *P*  0.05 from diabetic controls.

A modest nonsignificant decrease in NO levels in kidney of the diabetic control group was observed when compared to nondiabetic control group (Figure 4). However, treatment with* Withania coagulans *has significantly (*P* < 0.05) increased the NO levels in kidney as compared to the diabetic control (Figure 4).

### 3.7. Effect of* Withania coagulans* on Proinflammatory Cytokines

**Figure 5:**
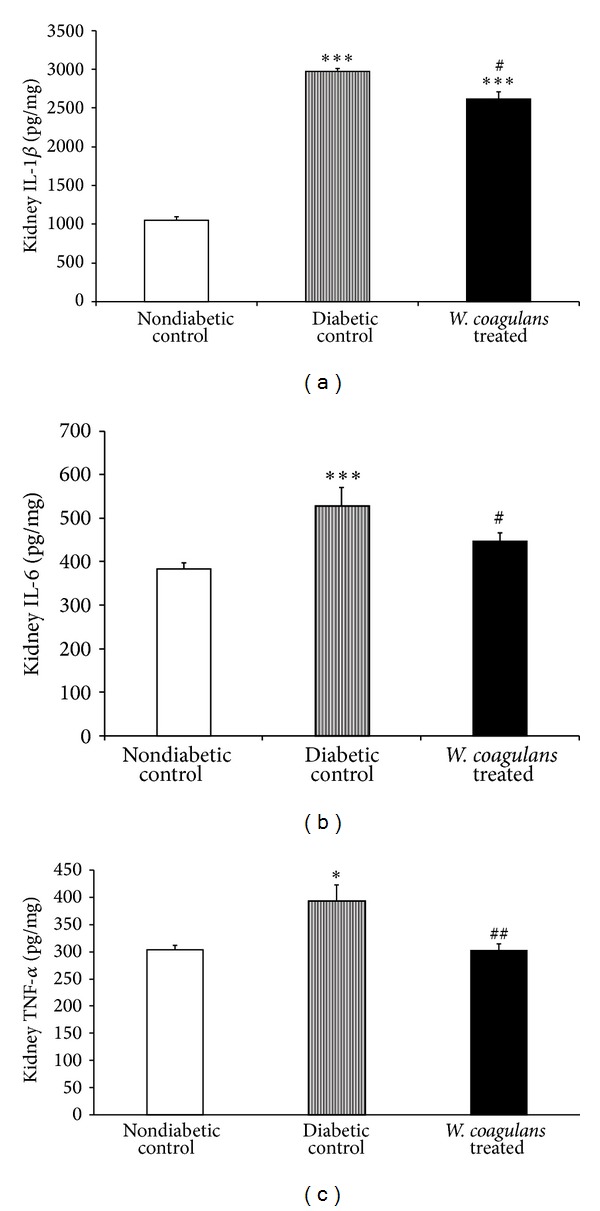
Effect of* Withania coagulans *on kidney levels of (a) IL-1*β*, (b) IL-6, and (c) TNF-*α*. Diabetic controls showed significantly elevated kidney IL-1*β* (a), IL-6 (b), and TNF-*α* (c) cytokines levels, compared to nondiabetic controls.* Withania coagulans* treatment significantly decreased the IL-1*β* (a), IL-6 (b), and TNF-*α* (c) compared to diabetic controls. Results are means ± SEM; *n* = 6 rats; *P < 0.05, ***P < 0.001 from nondiabetic controls; ^#^ *P*  0.05, ^##^ *P* < 0.01 from diabetic controls.

Figures 5(a)–5(c) represent the levels of kidney proinflammatory cytokines such as IL-1*β*, IL-6, and TNF-*α* of different experimental groups: nondiabetic control, diabetic control, and* Withania coagulans *treated. There was a significant increase in the level of IL-1*β* (*P* < 0.001), IL-6 (*P* < 0.001), and TNF-*α* (*P* < 0.05) in kidneys of STZ-induced diabetic rats when compared to nondiabetic control group. A significant decline in the kidney levels of IL-1*β* (*P* < 0.05), IL-6 (*P* < 0.05), and TNF-*α* (*P* < 0.01) was observed on treatment with* Withania coagulans *when compared to diabetic control.

### 3.8. Effect of* Withania coagulans* on Immunoregulatory Cytokines

**Figure 6:**
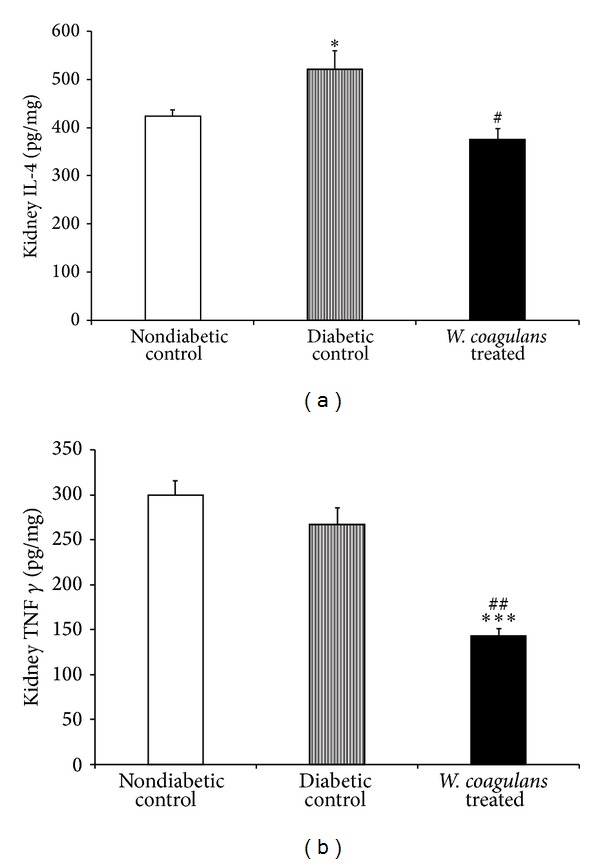
Effect of* Withania coagulans* on kidney levels of (a) IL-4 and (b) IFN-*γ*.* Withania coagulans* treatment significantly decreased the kidney IL-4 and IFN-*γ* compared to diabetic controls. Results are means ± SEM; *n* = 6 rats; *P < 0.05, ***P < 0.001 from nondiabetic controls; ^#^ *P*  0.05, ^##^ *P* < 0.01 from diabetic controls.

The levels of IL-4 and IFN-*γ* in kidneys of different experimental groups are presented in Figure 6. Though the change in IFN-*γ* levels was not altered significantly, a significant (*P* < 0.05) increase in the IL-4 level was observed in STZ-induced diabetic rats when compared to the nondiabetic control group. However, treatment with* Withania coagulans *extract has significantly reduced the levels of IL-4 (*P* < 0.05) and IFN-*γ* (*P* < 0.01) in kidneys as compared to diabetic rats.

## 4. Discussion

In the present study, STZ-injected rats show significant rise in plasma glucose level along with decrease in serum insulin and body weight and increase in kidney weight in comparison with nondiabetic control rats, indicating the development of diabetes as characterized by chronic and persistently elevated plasma glucose level. Decreased body weight in STZ-induced diabetic rats is believed to be due to dehydration, breakdown, and catabolism of fats and proteins. Increased catabolic reactions after STZ administration leads to muscle wasting and decreased body weight. STZ induces diabetes by selectively destroying insulin producing pancreatic endocrine cells and damages kidney similar to early stage diabetic nephropathy [20, 21]. This is in agreement with various other observations that STZ-induced animals exhibit diabetic renal complications [8, 9, 22]. However, treatment with* Withania coagulans *restored body weight, kidney weight, and reduced hyperglycemia, as well as enhancing survival and general body growth of diabetic rats. Ratio of kidney/body weight is an index of renal hypertrophy and a significant increase in kidney/body weight indicates renal injury in STZ administered rats. However, treatment with* Withania coagulans *to the diabetic rats has markedly reduced renal hypertrophy as evidenced by reduction of kidney/body weight when compared to the diabetic control. These results demonstrate that the extract of* Withania coagulans *exhibits antihyperglycemic effects through modulation of insulin and related enzyme activities in consonance with other studies demonstrated antihyperglycemic as well as protective effect in other organs apart from kidneys [15–17].

Pathogenic mechanisms underlying the progressive renal diseases in diabetics are known to be multifactorial including oxidative stress, inflammation, and immune-dysfunction [5, 6]. Oxidative stress ultimately triggers inflammation and modulates immunologic cascade in progression of renal damage from genesis to progression [2, 3]. Hyperglycemia-induced oxidative stress and inflammation unleash a cascade of events that affect cellular proteins, gene expression, and cell surface receptor expression, ultimately resulting in progressive pathologic changes in diabetic kidneys [4]. To counteract oxidative stress, the first line of defense against reactive oxygen species (ROS) is GSH, an intracellular nonprotein thiols compound, which also participate in second line of defense as a substrate or cofactor for GSH-dependent enzymes to detoxify ROS generated toxic byproducts and prevent propagation of free radicals [23]. In the present study, decreased levels of GSH in serum of STZ-injected rats might be explained by depletion or consumption of GSH in removing the hyperglycemia generated peroxides. Following treatment with* Withania coagulans*,the improvement in GSH level demonstrates its antioxidant activity in agreement with other studies where* Withania coagulans *was shown to ameliorate oxidative stress [15–17]. Although no significant change in renal GSH levels was observed in the STZ administered rats, a significant rise in kidney GSH levels was obtained following treatment with* Withania coagulans *indicating increased production of GSH.

Furthermore, ROS, by impairing antioxidant defense, renders the kidneys more susceptible to lipid peroxidation. ROS induced lipid peroxidation is a marker of cellular oxidative damage and is an important pathogenic event in renal injury [24]. In our study, increased level of lipid peroxidation product, MDA, clearly indicates oxidative stress in diabetic kidneys. Following treatment with* Withania coagulans*,the inhibition of lipid peroxidation as evidenced by decreased albeit not significant MDA levels in kidney demonstrates the antioxidant effect of* Withania coagulans *in agreement with previous studies which showed its antilipid peroxidation activity [15–17]. In addition to reduction of hyperglycemia, the ability of* Withania coagulans *to prevent GSH depletion and lipid peroxidation seems to be advantageous to mitigate the oxidative stress and may delay the development and progression of renal complications in diabetes.

In addition, change in MPO activity has been demonstrated to play role in degenerative and immunologic changes of the kidney [25]. In this study, we did not observe a significant change in MPO activity. Changes in renal NO levels have been linked to the pathogenesis of diabetes and associated complications [26]. The complex oxidative milieu in diabetes triggers several pathophysiologic mechanisms that simultaneously stimulate or suppress NO production at a given stage of the disease. Many studies demonstrated that decrease in renal NO levels are partly results of enhanced oxidative stress and partly of decreased NOS expression [27]. However, treatment of diabetic rats with* Withania coagulans *significantly increased NO levels in the kidneys. This effect is supported by the reduction of oxidative stress and could be ascribed to the induction of NOS following a counterbalance of NOS activity under the oxidative burst in accordance with previous other studies [21, 24].

Recent studies have shown that long-term, innate immune system activation resulting in chronic low grade inflammation is associated with the risk of developing renal complications, implying that immunologic and inflammatory mechanisms play a significant role in disease development and progression [4–6]. Studies suggest that proinflammatory cytokines (IL-1*β*, IL-6, and TNF-*α*) and IFN-*γ* (Th1) and IL-4 (Th2) act as pleiotropic polypeptides that are independently associated and exert an important diversity of actions in diabetic kidneys from development to progression [2, 6]. Both infiltrating immune cells (mainly monocytes and macrophages) and renal resident cells (endothelial, mesangial, dendritic, and epithelial) produce proinflammatory cytokines such as IL-1*β*, IL-6, and TNF-*α* [28]. The release of these cytokines may lead to renal injury through several mechanisms [6]. Being chemotactic in nature, the produced chemokines recruit more inflammatory cells and activate fibroblasts and matrix production, therefore, inducing the development of diabetic renal complications [2, 6]. Further, IFN-*γ* secreted by activated T cells and NK cells in conjunction with proinflammatory cytokines activates macrophages and stimulates chemokine production which result in pathological lesions of diabetic renal diseases. Increased IL-1*β* in kidney is known to increase the subsequent expression of chemotactic factors and adhesion whereas increased IL-6 levels are known to alter endothelial permeability, induce proliferation, and increase fibronectin expression [6, 9]. In the present study, a significant increase in cytokine levels, IL-1*β*, IL-4, IL-6, and TNF-*α*,, in kidneys of rats injected STZ are in agreement with previous studies [21]. Following treatment with* Withania coagulans*, significant reduction in the level of these cytokines is clearly suggestive of its anti-inflammatory effect in diabetic kidney. Thus, the attenuation of proinflammatory cytokines and lipid peroxidation along with diminution of hyperglycemia and improved antioxidants by* Withania coagulans *treatment is clearly suggestive of its beneficial effects in diabetic kidney.

Recent evidences in alternative medicine have encouraged that whole herb formulation is an effective therapeutic modality in chronic diseases including diabetes due to their multitudes of synergistic bioactivities and nutritional properties [29]. The current concept has revealed a new class of agents, known as adaptogens which increase resistance of the organism to aversive stimuli threatening to perturb internal homeostasis. The adaptogens have the potential to reverse stress induced immunity deregulation and organ dysfunction by sparing the antioxidants and modulating the immune system [29]. The immunoregulatory cytokines play an essential role in downmodulating adaptive and innate immune responses leading to chronic inflammation [4]. Several studies have demonstrated the adaptogenic activity of* Withania species* by inducing immune-surveillance [14]. In the present study, the decreased levels of immunoregulatory cytokines, IL-4 and IFN-*γ* are strongly suggestive of the immunomodulatory and associated adaptogenic potential of* Withania coagulans *in consonance with therapeutic benefits of adaptogenic medicines in chronic diseases [30].* Withania *described in Indian Ayurvedic medicine as Rasayana drugs is believed to produce its positive health impact through immune-enhancing, longevity promotion, and molecular nutritive effect [29].

Based on the present study findings and supportive data from ethnomedicinal, clinical, and preclinical studies [11, 15, 17–19],* Withania coagulans *holds promise for its potential in delaying the progression of renal complications in diabetes. Being a natural agent and due to its time tested use since ancient time is supportive of its relative safety. This is encouraging for* Withania coagulans *to be used in prevention and treatment of preventing renal complications in diabetes. Coupled with multiple pharmacological effects such as antihypertensive, hypolipidemic, hypoglycemic, immunosuppressive, antioxidant, anti-inflammatory, and adaptogenic activity,* Withania coagulans *might be a good therapeutic agent against renal complications of diabetes which involves multifactorial aetiopathogenesis.

To conclude, the results of our study demonstrate that treatment with* Withania coagulans *reduces the occurrence of oxidative stress and inflammation and improves hyperglycemia owing to its synergistic and polypharmacological properties. Further studies are encouraged for the translational application in humans.

## Figures and Tables

**Table 1:** Effect of *Withania coagulans* on weight changes of body and kidney to body weight ratio. Twenty-one-day treatment with *Withania coagulans * extract caused a significant improvement in the body weight and kidney to body weight ratio compared to diabetic controls.

Groups	Body weight (gms)		Kidney weight: body weight
	Before treatment	During treatment	
Nondiabetic controls	368.166 ± 17.20	419.33 ± 22∗∗∗	0.0029 ± 0.00012
Diabetic controls	266.4 ± 5.61	259.2 ± 5.39∗∗∗	0.00469 ± 0.00017∗∗∗
*W*. *coagulans* treated	269.57 ± 7.09	292 ± 12.49^∗∗,#^	0.00408 ± 0.000084^∗∗∗,#^

Results are means ± SEM; *n* = 6 rats; ∗∗*P* < 0.01, ∗∗∗*P* < 0.001 from nondiabetic controls; ^#^ *P* 0.05 from diabetic controls.
